# MiR-199a-3p inhibits proliferation and induces apoptosis in rheumatoid arthritis fibroblast-like synoviocytes via suppressing retinoblastoma 1

**DOI:** 10.1042/BSR20180982

**Published:** 2018-11-14

**Authors:** Yufan Wangyang, Linhong Yi, Tao Wang, Yanbo Feng, Guangwang Liu, Dongya Li, Xin Zheng

**Affiliations:** 1 Department of Orthopaedics, Xuzhou Central Hospital, No.199 Jiefang Road, Xuzhou 221009, China; 2 Department of Orthopaedics, Xuzhou Clinical School of Xuzhou Medical University, No.199 Jiefang Road, Xuzhou 221009, China; 3 Department of Orthopaedics, The Affiliated Hospital of Xuzhou Medical University, No.99 Huaihai Road West, Xuzhou 221006, China

**Keywords:** expression, fibroblast-like synoviocytes, miR-199a-3p, rheumatoid arthritis

## Abstract

**Background** Fibroblast-like synoviocytes (FLSs) that line the intimal synovium play a crucial role in the pathogenesis of rheumatoid arthritis (RA). miR-199a-3p is a highly conserved miRNA that has been shown to regulate a variety of growth behaviors in diverse cell types. However, the role of miR-199a-3p in RA-FLS is still unknown. **Methods** Here, we presented the first experimental evidence showing that miR-199a-3p was a critical regulator of RA-FLS function. **Results** miR-199a-3p expression was significantly reduced in RA-FLS compared with normal FLS. Ectopic expression of miR-199a-3p significantly inhibited RA-FLS proliferation and induced apoptosis, which was demonstrated by an increase in caspase-3 activity and Bax/Bcl-2 ratio. Our bioinformatics analysis identified Retinoblastoma 1 (*RB1*) gene to be a direct target of miR-199a-3p. In RA-FLS, miR-199a-3p directly targetted the 3′-UTR of *RB1* mRNA and suppressed endogenous RB1 expression, whereas miR-199a-3p-resistant variant of RB1 was not affected. Silencing RB1 decreased cell proliferation and promoted apoptosis in RA-FLS, an effect comparable with miR-199a-3p overexpression. Enforced expression of RB1 partially restored cell proliferation and attenuated apoptosis in miR-199a-3p-overexpressing RA-FLSs. **Conclusion** In summary, miR-199a-3p is down-regulated in RA-FLS, and miR-199a-3p inhibits proliferation and induces apoptosis in RA-FLS, partially via targetting RB1. The miR-199a-3p/RB1 pathway may represent a new therapeutic target for RA.

## Introduction

Rheumatoid arthritis (RA) is a symmetric autoimmune disease that causes chronic inflammation of the joints and other areas of the body. RA occurs when autoantibodies attack the synovium, the connective tissue lining of synovial joint surface, resulting in thickening of the synovial membrane and destruction of the cartilage and bone. RA can affect people of all ages. The cause of RA is unknown, although both inherited and environmental risk factors have been identified [1]. There are no cures for the cause of RA. Current treatment for RA is largely dependent on symptom management and surgery [2] reducing pain and inflammation. If RA is untreated or unresponsive to therapy, joint destruction will ultimately lead to escalating pain and deformity, loss of joint function, and often difficulties in maintaining daily activities and employment. Today, RA is one of the most common autoimmune disorders affecting 1% of the global population [3]. The development of efficacious RA therapies remains an urgent public health need.

Fibroblast-like synoviocytes (FLSs, also called type B synoviocytes) are a special type of mesenchymal-derived cells lining the internal synovium. FLSs display many markers of fibroblasts that show characteristics, which are distinct from other fibroblasts including secretion of lubricin, and expression of unique surface markers such as CD55, VCAM-1, cadherin-11, integrins, and their receptors. FLS is a crucial player in RA pathogenesis. RA-FLS directly participates in synovial hyperplasia and the production of cytokines that perpetuates local inflammation. RA-FLS also contributes to modulation of immune cells and proteolytic destruction of extracellular matrix, cartilage, and bone. Targetting RA-FLS has been recognized as a novel therapeutic approach with potentially improved clinical outcomes and less impact on systemic immunity [4].

miRNAs are a family of short (20–24 nts) non-coding RNAs that are involved in post-transcriptional regulation of gene expression. Recently, miR-199a-3p has been identified as a highly conserved miRNA that regulates a variety of growth behaviors in diverse cell types. Of note, miR-199a-3p has consistently displayed an anti-proliferative [5–8], anti-invasive [5,9,10], anti-inflammatory [11], and pro-apoptosis [6] effects in various cell types, which is believed to be therapeutically desirable for RA-FLS. However, the role of miR-199a-3p in RA-FLS has never been studied. This prompted us to explore the effect of this novel miRNA in RA-FLS. Here, we presented first evidence that miR-199a-3p is indeed dysregulated in RA-FLS, which is a critical regulator of RA-FLS growth behavior via targetting retinoblastoma 1 (*RB1*) gene.

## Methods

### Blood samples

All procedures related to patients were approved by the Ethics Committee of the Affiliated Hospital of Xuzhou Medical University and conformed to the Declaration of Helsinki. Written informed consent was obtained from all study subjects. Venous blood samples from healthy individuals (*n*=10) and RA patients (*n*=19) were collected at the Affiliated Hospital of Xuzhou Medical University from November 2012 to February 2014. Patients with confirmed severe liver or kidney diseases, malignancies, or acute heart failure were excluded. Plasma was isolated within 4 h of blood collection by centrifugation at 1500×***g*** for 15 min for RNA extraction.

### Cell culture and transfection

Normal human FLSs and RA-FLSs were purchased from Cell Applications (San Diego, CA, U.S.A.) and cultured in Dulbecco’s modified Eagle’s medium (DMEM) supplemented with 10% FBS, 100 U/ml penicillin and 100 mg/ml streptomycin at 37°C with 5% CO_2_. Transfection of DNA constructs and siRNA was performed using Lipofactamine 2000 (Invitrogen, Carlsbad, CA, U.S.A.) following the manufacturer’s instructions.

### DNA constructs and siRNA

To generate the miR-199a-3p overexpressing construct, a DNA fragment containing human miR-199a-3p precursor was amplified by PCR from human genomic DNA and inserted into a pSilencer 4.1-CMV puro mammalian expression vector (Thermo Fisher Scientific, Waltham, MA, U.S.A.). To create the RB1 overexpressing construct, the full-length human RB1 coding sequence (excluding 3′-UTR) was amplified by PCR and cloned onto a pcDNA3.1(+) mammalian expression vector (Invitrogen). To generate luciferase reporter constructs, the full-length RB1 3′-UTR was amplified by PCR and inserted into a pmirGLO dual-luciferase miRNA target expression vector (Promega, Madison, WI, U.S.A.), 3′ of the firefly luciferase reporter gene *luc2*, while the *Renilla* luciferase gene *hRluc* provides normalization. The predicted miR-199a-3p targetting site within RB1 3′-UTR was mutated by a PCR-based site-directed mutagenesis kit (Stratagene, La Jolla, CA, U.S.A.). miR-199a-3p mimic, siRNA for RB1 (si-RB1), and their respective controls were purchased from Ribobio, Guangzhou, China. siRNAs targetting two different sites of *RB1* gene and negative control siRNAs were synthesized by Shanghai GenePharma Co., Shanghai, China.

### Reverse transcription-quantitative real-time PCR

Total RNA was extracted from isolated plasma or cultured cells using TRIzol reagent (Invitrogen) per manufacturer’s instructions. For mRNA quantitation, cDNA was synthesized by PrimeScript RT reagent Kit (Takara, RR047A), and qPCR was performed using SYBR PrimeScript RT-PCR kit (Takara, Dalian, China). *GAPDH* was chosen as the reference gene for mRNA quantitation. For miRNA quantitation, miRNA was converted into cDNA by TaqMan MicroRNA Reverse Transcription Kit (Applied Biosystems, Foster City, CA, U.S.A.). qPCR was performed using TaqMan Human MicroRNA Assay Kit (Applied Biosystems), and miRNA expression was quantitated by normalization to small nuclear RNA gene *U6*.

### Cell proliferation assays

Cell proliferation was assayed by several complementary assay methods, including the MTT assay, the Cell Counting Kit-8 (CCK8) assay, and the ErdU assay.

For the MTT assay, cells were seeded on to 96-well plates at 5 × 10^3^ cells/well and cultured for 48 h. The MTT solution (0.5 mg/ml; Sigma–Aldrich, St. Louis, MO, U.S.A.) was added into each well followed by incubation for 4 h at 37°C. DMSO was then added to dissolve the crystals and absorbance at 570 nm wavelength was measured.

The cell growth curve was generated by the CCK8 assay performed at 24-h intervals following the manufacturer’s protocol. In brief, transfected cells were plated on 96-well plates at 3 × 10^3^ cells/well, and 10 μl/well CCK8 (Promega) solution was added to separate wells every 24 h, incubated for 2 h, and measured spectrophotometrically at 450 nm.

The ErdU assay was conducted using a Cell Light EdU DNA imaging kit (Guangzhou RiboBio). Briefly, cells were transfected on 96-well plates. Forty-eight hours after transfection, 5-ethynyl-20-deoxyuridine (EdU) (100 mM) was added to each well, and the cells were cultured for an additional 2 h. The cells were then stained as follows: decant the medium, add 4% paraformaldehyde in PBS to fix cells at room temperature for 30 min, wash with 2 mg/ml glycine in PBS for 5 min on an orbital shaker, add 0.2% Trion X-100/PBS for 10 min, wash with PBS for twice, add click reaction buffer (100 mM Tris/HCl, pH 8.5; 1 mM CuSO_4_, 100 mM Apollo 550 fluorescent azide, and 100 mM ascorbic acid) and incubate for 10–30 min protected from light, wash with 0.5% Triton X-100/PBS for three times, stain with Hoechst (5 mg/ml) for 30 min at room temperature, wash with 0.5% Triton X-100/PBS for five times, and finally add 150 ml/well PBS. Cells were imaged and analyzed on a BD Pathway 855 high-content analysis system (BD, San Jose, CA, U.S.A.). EdU-positive percentage was calculated as: (EdU+ cells)/(Hoechst-stained cells) × 100%.

### Luciferase reporter assay

Cells were seeded on to 24-well plates at a density of 1 × 10^5^ cells/well and co-transfected with the luciferase report constructs and pSilencer 4.1-miR-199a-3p or pSilencer 4.1-CMV puro plasmid (no-insert control) (0.2 μg/well each). A *Renilla* luciferase expressing pRL-TK vector (Promega) was used to control for transfection efficiency. At 24 h after transfection, cells were lysed and measured for luciferase activities using the dual-luciferase reporter assay system (Promega). The activity of firefly luciferase was normalized to that of *Renilla* luciferase.

### Caspase-3 activity assay

Cellular caspase-3 activity was quantitated using a colorimetric assay kit according to the manufacturer’s instructions (BioVision, Mountain View, CA, U.S.A.). Absorbance was recorded at a wavelength of 405 nm.

### Apoptosis assay

Apoptosis was determined by FITC Annexin V Apoptosis Detection Kit I (BD Pharmingen, San Diego, CA, U.S.A.) in conjunction with a vital dye propidium iodide (PI), following the manufacturer’s instructions. Briefly, cells were detached, washed, and stained with FITC Annexin V and PI for 15 min protected from light. After washing, FITC and PI staining was examined by flow cytometry (BD Biosciences, San Jose, CA, U.S.A.).

### Western blot

Cells were lysed in radioimmunoprecipitation assay buffer (Beyotime, Nantong, China) containing protease inhibitors (Sigma). Normalized protein samples (50 μg/lane) were resolved by SDS/PAGE and transferred on to nitrocellulose membranes. The following primary antibodies were used for the present study: anti-RB1 monoclonal antibody (Abcam, ab24, Cambridge, MA, U.S.A.), rabbit anti-caspase-3 polyclonal antibody (ab90437, Abcam), rabbit anti-poly (ADP-ribose) polymerase (PARP) polyclonal antibody (ab194586, Abcam), mouse anti-Bcl-2 monoclonal antibody (#15071, Cell Signaling Technology, Danvers, MA, U.S.A.), rabbit anti-Bax polyclonal antibody (#2772, Cell Signaling Technology), and anti-GAPDH monoclonal antibody (Beyotime). HRP–conjugated secondary antibodies were obtained from Santa Cruz Biotechnology (Dallas, TX, U.S.A.). Protein bands were visualized with the chemiluminescent system (Cell Signaling Technology). Densitometry was performed using Quantity One software (Bio-Rad Laboratories, Hercules, CA, U.S.A.).

### Statistics

Values were presented as means ± S.D. Statistical differences were determined using the Student’s *t* test for two groups, and one-way ANOVA with Tukey’s post hoc test for three or more groups. *P-*value <0.05 was considered as statistically significant.

## Results

### Expression of miR-199a-3p and RB1 was dysregulated in RA-FLSs

**Figure 1:**
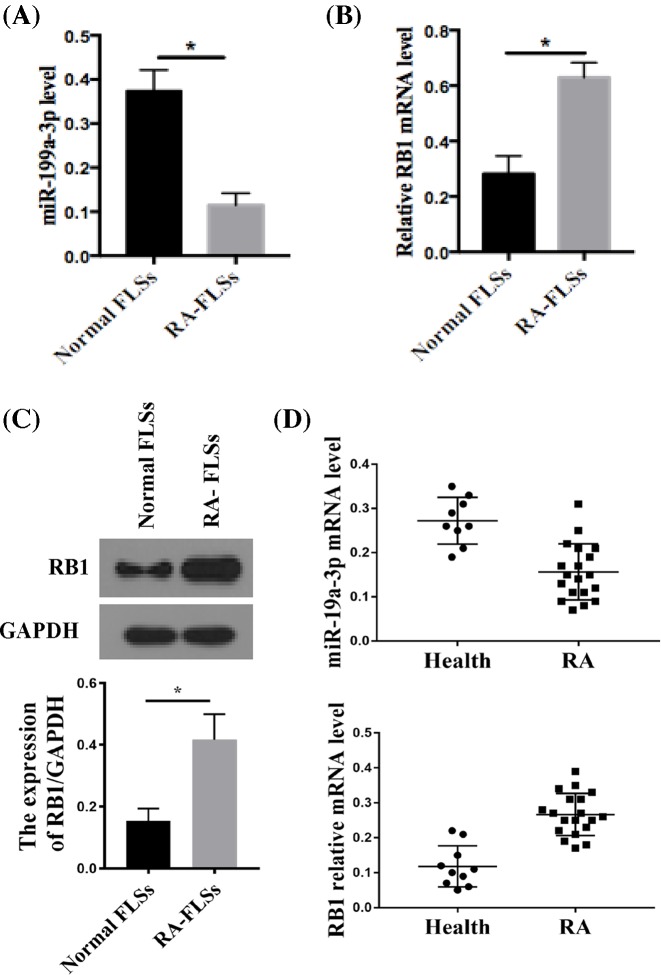
Expression of miR-199a-3p and RB1 was dysregulated in RA-FLSs miR-199a-3p (**A**) and RB1 (**B**) expression in normal human FLSs and RA-FLSs was measured by RT-qPCR and quantitated. *n*=6; *P<0.05 compared with normal FLSs. (**C**) RB1 protein expression in normal human FLSs and RA-FLSs was quantitated by Western blot. GAPDH was the loading control. *n*=6; *P<0.05 compared with normal FLSs. (**D**) Circulating miR-199a-3p and RB1 level in healthy (*n*=10) and RA (*n*=19) plasma was measured by RT-qPCR and quantitated. *P<0.05 compared with healthy plasma.

We first determined the expression level of miR-199a-3p and *RB1* mRNA in normal and RA-FLSs by RT-qPCR. Compared with normal FLSs, RA-FLSs showed significantly lower miR-199a-3p (Figure 1A) and significantly higher *RB1* mRNA expression (Figure 1B). The expression of RB1 protein was higher in RA-FLSs than normal FLS (Figure 1C). These data suggest that miR-199a-3p and RB1 expression is dysregulated in RA-FLSs.

We also measured the circulating miR-199a-3p and *RB1* mRNA expression levels in plasma. Circulating miR-199a-3p and *RB*1 mRNA level profiles followed the similar trend seen in FLS, with lower circulating miR-199a-3p and higher circulating RB1 expression in RA patient plasma compared with healthy individuals (Figure 1D).

### miR-199a-3p suppressed RA-FLS proliferation

**Figure 2:**
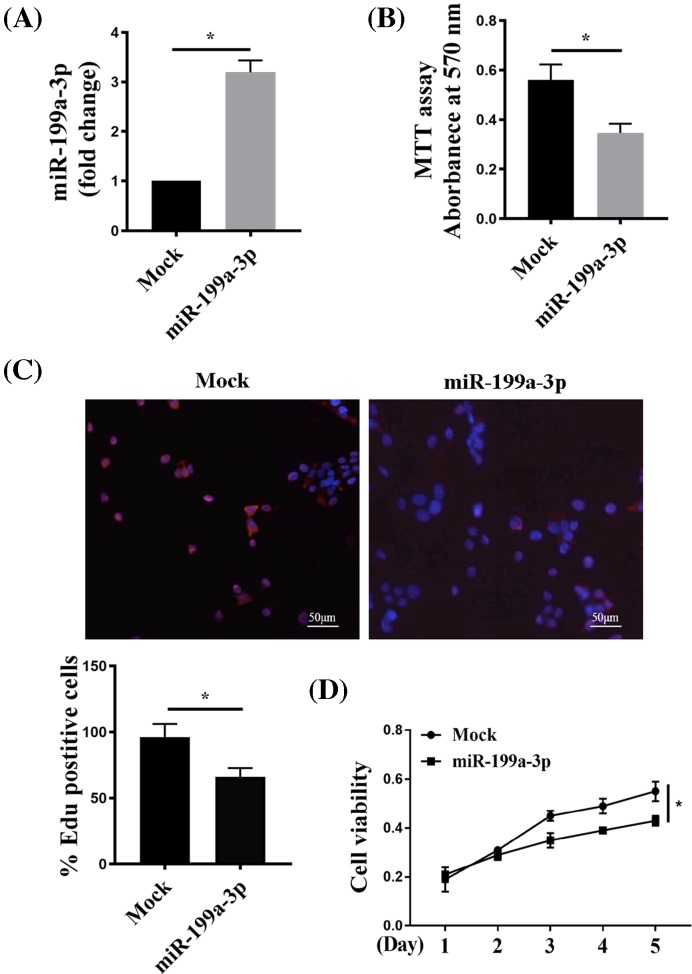
miR-199a-3p suppressed RA-FLS proliferation (**A**) RT-qPCR of miR-199a-3p in RA-FLSs transfected with pSilencer 4.1-miR-199a-3p plasmid (miR-199a-3p) or no-insert control pSilencer 4.1-CMV puro plasmid (Mock). (**B**) Forty-eight hours post transfection, MTT assay was performed to test the viability of miR-199a-3p or Mock RA-FLSs. (**C**,**D**). EdU assay of miR-199a-3p or Mock transfected RA-FLSs 48 h post transfection. Cells were stained for EdU and Hoechst (to mark nuclei) (C), and quantitated for EdU+ cell percentage (D). CCK8 assay showed that ectopic expression of miR-199a-3p significantly inhibited RA-FLS proliferation rate over 5 days. *n*=4 or *n*=6 (A,D); *P<0.05 compared with Mock.

To elucidate the function of miR-199a-3p in RA-FLS, we restored its expression in RA-FLSs by delivery of miR-199a-3p overexpressing construct pSilencer 4.1-miR-199a-3p, and compared with cells receiving no-insert control pSilencer 4.1-CMV puro plasmid (mock). RT-qPCR confirmed marked increase in miR-199a-3p level in RA-FLSs transfected with pSilencer 4.1-miR-199a-3p (Figure 2A).

We measured the effect of miR-199a-3p overexpression upon cell proliferation via a few complementary methods. MTT assay showed that compared with mock, miR-199a-3p delivery significantly inhibited the proliferation of RA-FLSs after culturing for 48 h (Figure 2B). Similarly, EdU assay showed significantly decreased percentage of EdU+ RA-FLSs from miR-199a-3p overexpression comparing with mock (Figure 2C). To better determine the difference in cell proliferation, we traced cell growth every 24 h over 5 days via a less toxic CCK8 assay. Overexpression of miR-199a-3p significantly inhibited proliferation rate in RA-FLS over the 5-day course (Figure 2D). Together, these data suggested that miR-199a-3p suppressed RA-FLS proliferation.

### miR-199a-3p induced RA-FLS apoptosis

**Figure 3:**
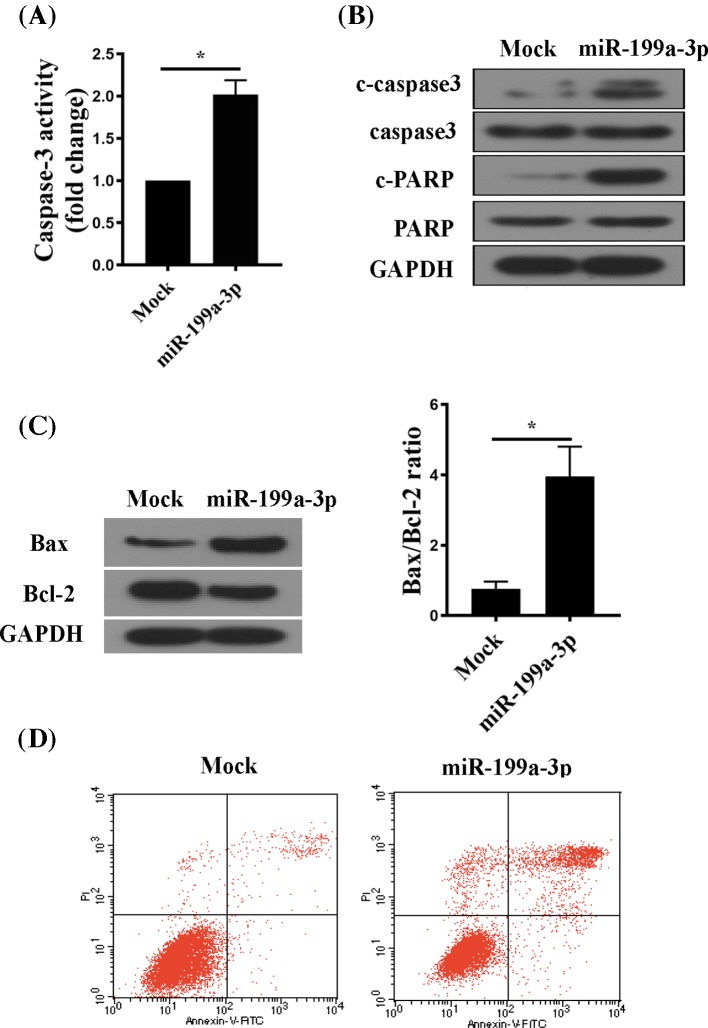
miR-199a-3p induced RA-FLS apoptosis (**A**) Caspase-3 activity was measured by a colorimetric method. (**B**) Western blot of cleaved caspase-3 (C-caspase-3) and cleaved PARP (C-PARP). GAPDH was the loading control. (**C**) Western blot of Bax and Bcl-2 protein (left) and quantitation of the Bax/Bcl-2 ratio (right). GAPDH was the loading control. (**D**) Summarized data of cell apoptosis (positive for Annexin V and PI) determined by flow cytometry in RA-FLS transfected with mock or miR-199a-3p. *n*=6; *P<0.05 compared with Mock.

The decreased MTT and CCK8 signal (Figure 2) could be attributed to changes in either proliferation or apoptosis. Next, we examined the effect of miR-199a-3p on apoptosis of RA-FLSs. Caspase-3 activity was greater in miR-199a-3p-overexpressing RA-FLSs than in controls (Figure 3A). Western blot analysis confirmed enhanced cleavage of caspase-3 and PARP in RA-FLSs after overexpression of miR-199a-3p (Figure 3B). In addition, the level of the pro-apoptotic protein Bax was increased and that of the anti-apoptotic protein Bcl-2 was decreased by overexpression of miR-199a-3p (Figure 3C). The Bax/Bcl-2 ratio was higher in miR-199a-3p-overexpressing RA-FLSs than that in controls (Figure 3C). Finally, flow cytometry analysis of apoptotic markers confirmed increased percentage of cell apoptosis in miR-199a-3p-overexpressing RA-FLSs (Figure 3D). Taken together, these results suggested that miR-199a-3p induced apoptosis in RA-FLSs.

### miR-199a-3p directly suppressed *RB1* gene expression

**Figure 4:**
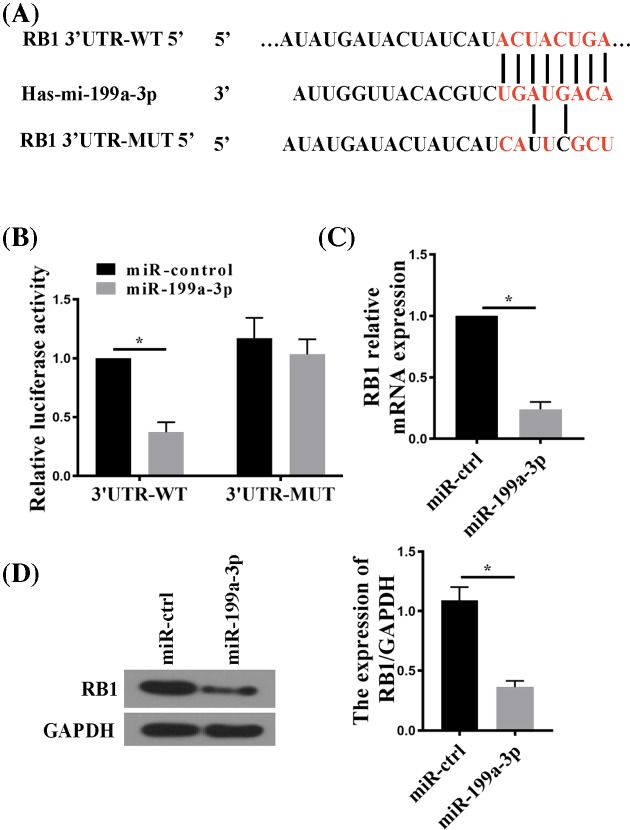
RB1 is a direct target of miR-199a-3p (**A**) TargetScan analysis showing the WT 3′-UTR of *RB1* mRNA containing a putative miR-199a-3p target site. A mutant (MUT) sequence was designed accordingly to be tested for luciferase assay together with the WT. (**B**) Luciferase reporter assay comparing WT with MUT RB1 3′-UTR targetting by miR-199a-3p. RA-FLSs cells were co-transfected with RB1 3′-UTR firefly luciferase reporter constructs harboring WT or MUT miR-199a-3p-targetting sequences and an miR-199a-3p-expressing plasmid (miR-199a-3p) or a pSliencer 4.1-CMV puro vector (miR-control). Firefly luciferase activity was normalized to Renilla luciferase activity. (**C**) RT-qPCR analysis of RB1 mRNA in RA-FLSs transfected with miR-199a-3p or miR-control. (**D**) RB1 protein expression in RA-FLSs transfected with miR-199a-3p or miR-control. *n*=5; *P<0.05 compared with miR-control.

In an attempt to search for the direct target genes of miR-199a-3p, we identified a predicted miR-199a-3p target site within the 3′-UTR of *RB1* mRNA using the TargetScan algorithm (http://www.targetscan.org) (Figure 4A). To validate the targetting of RB1 by miR-199a-3p, we generated luciferase reporter constructs harboring either the wild-type (WT) or mutant (MUT) form of RB1 3′-UTR containing the predicted miR-199a-3p-binding site (Figure 4A), and co-transfected each luciferase reporter construct with pSilencer 4.1-miR-199a-3p (miR-199a-3p) or pSilencer 4.1-CMV puro plasmid (miR-control). Luciferase reporter assay in transfected cells showed that in the presence of miR-199a-3p, the reporter containing WT miR-199a-3p-binding site showed significantly reduced luciferase activity, but not in the presence of miR-control (Figure 4B). The inhibitory effect of miR-199a-3p on the luciferase reporter activity was abolished when the predicted miR-199a-3p site was mutated. Consistent with the luciferase reporter assay, transfection with pSilencer 4.1-miR-199a-3p reduced the endogenous level of RB1 in RA-FLSs at both mRNA and protein levels (Figure 4C,D). In sum, these results suggest that miR-199a-3p directly suppressed RB1 expression by targetting its 3′-UTR.

### RB1 suppression inhibited proliferation and induced apoptosis in RA-FLSs

**Figure 5:**
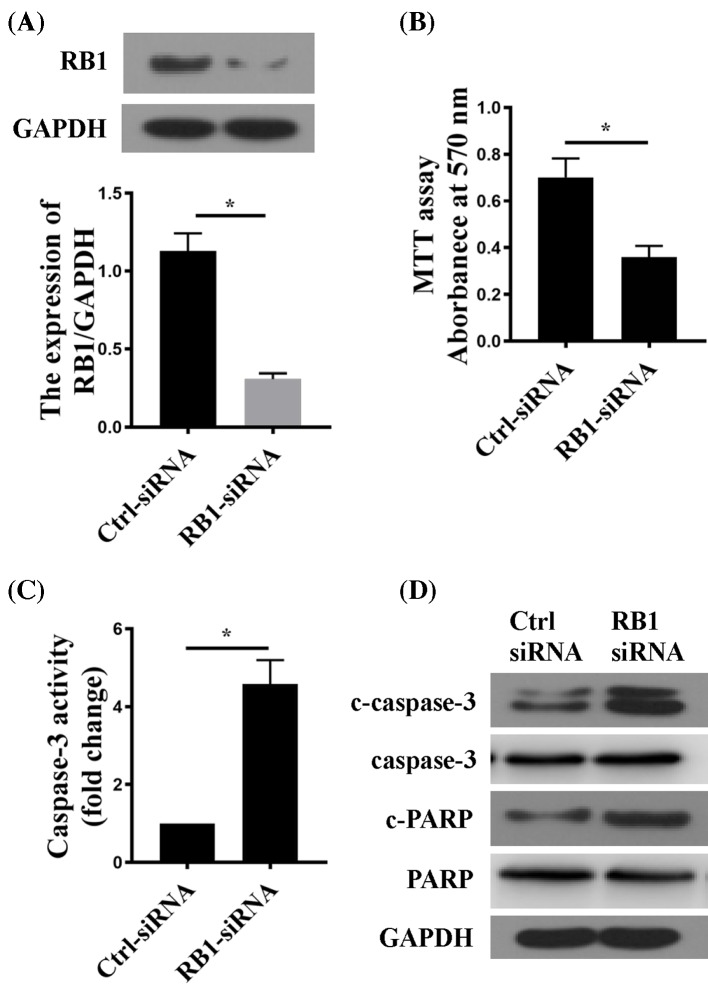
Down-regulation of RB1 inhibited cell proliferation and induced apoptosis in RA-FLS (**A**) RB1 protein expression in RA-FLSs transfected with control (Ctrl-siRNA) or RB1-targetting siRNA (RB1-siRNA). (**B**) MTT assay in siRNA-transfected cells after culturing for 48 h. (**C**) Caspase-3 activity in siRNA-transfected cells after culturing for 48 h. (**D**) Western blot of C-capase-3 and C-PARP protein in siRNA-transfected RA-FLSs. GAPDH was the loading control. *n*=6; *P<0.05 compared with Ctrl-siRNA.

To test the potential effects of RB1 suppression on RA-FLS, we knocked down RB1 expression by siRNA, and examined proliferation and apoptosis in RA-FLS. The efficiency of RB1-siRNA was confirmed by Western blot (Figure 5A). RB1 suppression significantly reduced RA-FLS proliferation and significantly activated pro-apoptotic proteins, as demonstrated by the reduced MTT signal (Figure 5B), enhanced caspase-3 activity (Figure 5C), and increased protein expression of cleaved caspase-3 and PARP (Figure 5D) after RB1 down-regulation.

### miR-199a-3p regulated RA-FLS proliferation and apoptosis partially via suppressing RB1

**Figure 6:**
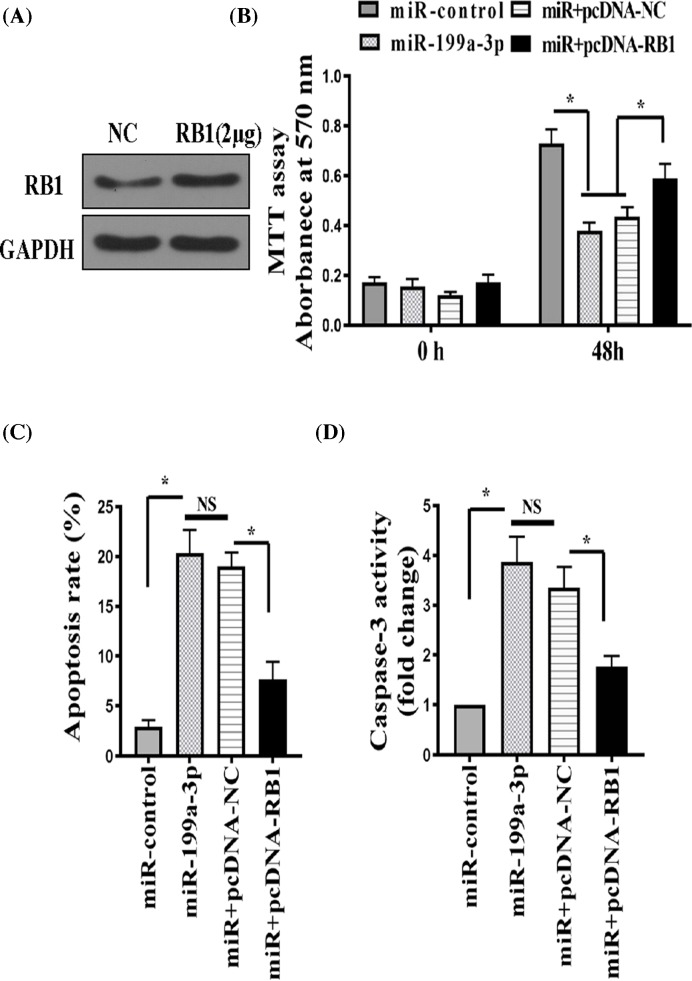
miR-199a-3p regulated RA-FLS proliferation and apoptosis partially via suppressing RB1 RA-FLS was transfected with an miR-199a-3p-expressing plasmid (miR-199a-3p) or a pSliencer 4.1-CMV puro vector (miR-control) or miR-199a-3p in combination with an RB1-overexpression plasmid (miR + pcDNA-RB1) or miR-199a-3p in combination with a negative control plasmid (miR + pcDNA-NC). (**A**) Western blot of RB1 protein in RA-FLSs transfected with negative control (NC) or pcDNA3-RB1. (**B**) Proliferation of transfected RA-FLS was measured by the MTT assay. (**C**) Apoptosis was measured by FITC Annexin V and PI staining followed by flow cytometry. (**D**) Caspase-3 activity was measured by a colorimetric method. *n*=6; *P<0.05. Abbreviation: NS, not significant.

Given the effects of RB1 suppression on RA-FLS proliferation and apoptosis, we next investigated whether RB1 could mediate the growth regulatory activity of miR-199a-3p in RA-FLSs. To this end, we explored the consequence of miR-199a-3p overexpression with or without RB1 overexpression in RA-FLSs. RB1 overexpression in RA-FLSs was clearly detected by Western blot (Figure 6A). Similar to Figure 2, miR-199a-3p overexpression alone significantly reduced RA-FLS proliferation as measured by MTT assay (Figure 6B), and increased RA-FLS apoptosis as measured by flow cytometry (Figure 6C) and caspase 3 activation (Figure 6D). Concomitant overexpression of RB1 partially reversed miR-199a-3p’s effect on the proliferation and apoptosis of RA-FLS (Figure 6B–D). While overexpression of RB1 fails to fully abolish miR-199a-3p’s effect, probably due to the already high expression level of miR-199a-3p, these data strongly suggest that RB1 is a target of miR-199a-3p, and miR-199a-3p regulates proliferation and apoptosis of RA-FLS, at least partially, by directly targetting RB1.

## Discussion

RA is a chronic autoimmune disease characterized by synovial inflammatory of the joints. RA-FLS plays a central role in the progression of chronic inflammation, partially by transforming from a quiescent phenotype into a highly proliferative, invasive, and destructive phenotype common to cancerous cells [12]. miR-199a-3p has been identified as a potent miRNA that consistently attenuated aggressive behaviors in cancer and other cell types, representing a potentially promising target in modulating RA-FLS function.

The major finding of our study is that miR-199a-3p was indeed a critical regulator of RA-FLS growth behavior. miR-199a-3p expression was reduced in RA-FLS and RA plasma compared with normal FLS or plasma. miR-199a-3p inhibited RA-FLS proliferation and induced apoptosis. Importantly, we identified *RB1* mRNA to be a direct target of miR-199a-3p, which at least partially mediated miR-199a-3p’s effect on RA-FLS proliferation and apoptosis.

Our study found miR-199a-3p expression was significantly reduced in RA-FLS compared with tumors [8,13–17] and inflammatory processes [11]. Recent studies all pointed to an inflamed and hypoxic milieu in RA synovium, which played an important role in the phenotypic transformation of FLS [4]. Of note, miR-199a-3p expression displays responsiveness to hypoxia [18] and pro-inflammatory cytokines [19], which may partially account for its aberrant expression in RA-FLS. In addition, the aberrant circulating level of miR-199a-3p and *RB1* mRNA in RA patient plasma, which conforms to the same trend detected in RA-FLS, demonstrated a potential clinical usefulness of circulating miR-199a-3p and RB1 for RA monitoring.

Our study provided the functional basis for leveraging miR-199a-3p for RA diagnosis or treatment. Of note, circulating miR-199a-3p level [20–22] or its target CD44 [23] have shown promising diagnosis or prognosis values in cancer. The current results suggest that miR-199a-3p is also a critical regulator of RA-FLS aggressiveness. The value of miR-199a-3p and/or RB1 expression as a biomarker for RA progression warrants further study, and developing strategies to target miR-199a-3p may represent a novel approach to improve the clinical outcome of RA.

Our study identified *RB1* mRNA as a direct target of miR-199a-3p. Mutation or aberrant expression of oncogene and tumor suppressor genes, such as TP53, have been shown in RA-FLS, but their role on RA pathophysiology has been unclear and controversial [24]. Our results provided direct functional relevance of RB1 in RA-FLS: it promotes RA-FLS proliferation and inhibits RA-FLS apoptosis, an effect that was under direct regulation of miR-199a-3p.

Our study has several limitations. First, comprehensive clinical and pathological surveys from RA patients and data from more clinical RA synovial tissues are required to firmly establish the expression profile of miR-199a-3p in RA-FLS. Second, in addition to RB1, there may exist other target genes and pathways contributing to cell proliferation and apoptosis phenotypes downstream of miR-199a-3p, as implied by other studies [23,25,26]. At last, whether miR-199a-3p affect other aggressive RA-FLS phenotype, such as migration, invasion, and expression of inflammatory markers, requires further study.

In conclusion, our study identified miR-199a-3p inhibited proliferation and induced apoptosis in RA-FLS via suppressing *RB1* mRNA. The miR-199a-3p/RB1 pathway may represent novel diagnostic and therapeutic opportunities for RA.

## Abbreviations

CCK8: cell counting kit-8
EdU: 5-ethynyl-20-deoxyuridine
FLS: fibroblast-like synoviocyte
PARP: poly (ADP-ribose) polymerase
PI: propidium iodide
RA: rheumatoid arthritis
RB1: retinoblastoma 1
RT-qPCR: reverse transcription-quantitative real-time PCR
